# Domestic accidents due to children’s falls: a cross-sectional study

**DOI:** 10.1590/1980-220X-REEUSP-2024-0192en

**Published:** 2024-10-21

**Authors:** Abiúde Nadabe e Silva, Lídya Tolstenko Nogueira, Ana Roberta Vilarouca da Silva

**Affiliations:** 1 Universidade Federal do Piauí, Programa de Pós-Graduação em Enfermagem, Teresina, PI, Brazil.

**Keywords:** Accidental Falls, Accidents, Home, Child, Preschool, Nursing, Accidentes por Caídas, Accidentes Domésticos, Preescolar, Enfermería

## Abstract

**Objective:**

To describe the occurrence of domestic accidents due to children’s falls.

**Method:**

This is a descriptive, cross-sectional study carried out with 181 parents and/or other caregivers of children aged between 2 and 5 years. Data were collected in March and April 2024, through a questionnaire containing 23 items, which were subjected to descriptive analysis.

**Results:**

The sample was composed mostly of mothers (93.9%), with a predominance of female children (52.2%), and 86.2% of infants suffered some type of fall at home. Falls from bed (56.4%), hitting the head/face (52.6%), mainly causing cuts/lacerations (16%), were predominant.

**Conclusion:**

The results show that it is essential to raise awareness among families about the imminent responsibility of protecting children from domestic accidents, protecting them from injuries that can be avoided through changes in behavior and adopting a proactive stance by intervening in anticipation of risk factors.

## RESUMEN

**Objetivo:**

Describir la ocurrencia de accidentes domésticos por caídas infantiles.

**Método:**

Se trata de un estudio descriptivo, transversal, realizado con 181 padres y/u otros cuidadores de niños con edades entre 2 y 5 años. Los datos fueron recolectados en marzo y abril de 2024, mediante un cuestionario que contenía 23 ítems, los cuales fueron sometidos a análisis descriptivo.

**Resultados:**

La muestra estuvo compuesta mayoritariamente por madres (93,9%), con predominio de niñas (52,2%), sufriendo el 86,2% de los infantes algún tipo de caída en el hogar. Predominaron las caídas desde la cama (56,4%), golpes en la cabeza/rostro (52,6%), provocando principalmente cortes/laceraciones (16%).

**Conclusión:**

Los resultados muestran que es fundamental concientizar a las familias sobre la inminente responsabilidad de proteger a los niños de los accidentes domésticos, protegerlos de lesiones que pueden evitarse mediante cambios de comportamiento y adoptar una postura proactiva interviniendo de manera anticipada en los factores de riesgo.

## INTRODUCTION

Childhood is characterized by different stages of development, with early childhood standing out – children up to 6 years of age. It is during this phase that most skills develop, such as speaking, walking, running, jumping and the ability to learn^(1)^. Therefore, as children grow, their curiosity is awakened and becomes a constant in their daily lives, which increases the risk of suffering accidents^(2)^.

In this way, accidents have contributed to increasing the rate of infant morbidity and mortality, since they are closely related to the childhood phase itself, in addition to often reflecting the family’s lack of protective capacity and the lack of knowledge of risk situations that permeate children’s daily life^(3)^.

In this regard, Primary Healthcare workers, by working in communities and maintaining closer contact with families, can develop educational actions to prevent accidents, especially during home visits. Therefore, it is necessary to assess safety aspects in the home environment, guide those responsible about simple measures for child safety, carry out local educational campaigns and train healthcare professionals, considering risk factors, vulnerabilities and the stage of child development^(4)^.

An accident is defined as an unintentional but preventable event that can occur at home, at work, in traffic, at school, in sports and leisure areas, and cause physical and/or emotional injuries^(5)^. Accidents are caused by several predictable factors, combined with people’s physical and psychological conditions as well as the structure present in the physical, social, cultural and organizational environment^(6)^. A fall, in turn, consists of an event in which a person inadvertently falls to the ground, floor or another level below the one at which they were^(7)^.

Thus, it is understood that there are several factors that trigger accidents, such as housing conditions, socioeconomic level, safety conditions of the external environment, lack of suitable places for recreation, lack of information on accident prevention, child psychological and physical characteristics, among others^(3)^. In addition to causing social, economic and emotional costs, accidents also cause consequences that affect families and society, affecting many children^(8)^.

Accidents involving falls involving children occur in and around the home, in schools, in playgrounds, during sports and leisure activities, and outdoors^(7)^. Between 2000 and 2019, falls accounted for approximately 31,818 deaths of children and adolescents under 15 years of age worldwide^(9)^.

Data from the Viva Survey regarding the general characteristics of care for accidental falls in emergency and urgent care services in 20 Brazilian state capitals and the Federal District revealed that 57.3% of falls occurred at the victim’s home, including falls from the same level (57.2%), stairs or steps (18.3%), beds, cribs, hammocks or other furniture (10.4%), scaffolding, trees, roofs or slabs (4.8%), among others^(10)^.

Accidental falls caused injuries such as contusions, sprains or dislocations (59.3%), trauma (20.2%) and cuts or lacerations (14.2%), affecting the head or neck (23.4%), trunk (14.3%), upper limbs (28.5%) and lower limbs (33.8%), with 9.1% requiring hospitalization. Care for accidental falls at home was more frequent among the extreme age groups of 0 to 9 years (71.6%) and 60 and over (71.8%)^(10)^.

A study carried out in Ceará revealed that most domestic accidents involving young children occurred in low-income populations, with average levels of education, young mothers and with several people living in the same house, with falls being the most prevalent type of accident (66.6%)^(11)^. Another study carried out in Brazil showed that the majority of falls involving children occurred at home (60.7%), with 52.3% of accidents occurring with children aged 0 to 5 years^(12)^.

In this context, nursing has an important responsibility in aspects involving children’s health, especially in accident prevention, since caregivers need to be made aware of and guided regarding environmental risks, in order to promote behavioral changes in the way they provide care and avoid episodes of falls^(13)^.

Although national and international literature present information about the profile of domestic accidents due to falls in childhood, it is understood that each region has its own nuances, which is why this study is necessary in order to present a situational diagnosis of the location of interest in order to then design prevention measures according to the needs evidenced. Furthermore, after manually searching for articles published in the last five years in some Brazilian journals, it was observed that the scope of studies on this topic is limited.

Therefore, by identifying the main types of falls, this study provides knowledge that can guide the development of educational programs and materials to instruct families on how to prevent children from falling at home, intervening, above all, in modifiable risk situations. Thus, the present study aimed to describe the occurrence of domestic accidents due to children falling.

## METHOD

### Study Design

This is a descriptive, cross-sectional study. Data reporting followed the STrengthening the Reporting of Observational studies in Epidemiology (STROBE) recommendations^(14)^. This research derives from a multi-method study entitled “*Evidências de validade de cartilha educativa para a prevenção de acidentes domésticos por queda infantil*”.

### Site

The study was carried out in Valença do Piauí, Piauí, located 210 km from the capital, Teresina, with a territorial area of 1,333,722 km^2^, 22,279 inhabitants and a Human Development Index (HDI) of 0.647, with the mean monthly wage of formal workers being 1.6 minimum wages^(15)^.

In this site, there are four municipal public schools and three private schools, which assist children between 2 and 5 years old. Thus, a draw was made to select two public schools and one private school. It was decided to conduct research only in the urban area, due to the geographical distance and difficulty of access to communities in rural areas.

### Population, Inclusion and Exclusion Criteria

The study population consisted of parents and/or other caregivers of 340 children, 138 from school A, 117 from school B and 85 from school C. It should be noted that, for each child, only one caregiver was surveyed, whether the father, mother or guardian.

Eligibility criteria for establishing study participants were being a father, mother or other caregiver of children between 2 and 5 years of age, and being of legal age, i.e., being 18 years of age or older. Exclusion criteria were being a father, mother or other caregiver with any cognitive or behavioral difficulties, severe visual and/or hearing impairment that made it impossible to read, understand and complete the questionnaire as well as caregivers of children who had severe motor disabilities.

Consequently, two deaf couples – mothers and fathers – were excluded from the study due to the absence of a professional sign language interpreter who could convey the information in an understandable manner.

It is worth noting that four caregivers, who self-declared as illiterate, expressed interest in participating in the research. In this context, it was understood that, although they could not read or write, they were able to hear the questions and express their experiences verbally. With this understanding, the researcher read the questionnaire audibly to each illiterate participant individually and completed it according to the answers reported orally, and they were therefore included in the sample.

Such field diary reports are relevant, as they reveal the population’s different needs and encourage researchers to seek alternative educational resources and research instruments, in order to carry out health and safety promotion activities that are accessible to everyone.

### Sample Calculation

To calculate the sample, the formula for a finite population was used, stratified by proportion, with a 5% sampling error and a 95% confidence level^(16)^, which resulted in a minimum sample of 180.61 ≅ 181 participants.

### Data Collection

The data were collected by the main researcher in March and April 2024, after prior contact with the principals of each school, who proceeded promptly and agreed that data collection would be carried out at the time when parents and/or other caregivers were dropping off their children at school. Thus, the researcher gathered study participants in groups of 30 people, in a reserved room at the schools, in the morning and afternoon shifts, on alternate days, until reaching sample size.

It is important to note that managing each school informed parents and/or caregivers in advance – via message in WhatsApp^®^ groups of each class – that the research nurse would be at the school in the following days to conduct the research. Even so, in both educational institutions, many people refused to answer the questionnaire, claiming lack of time and/or not wanting to participate in the study, despite the researcher having explained, in person, the objectives and benefits of the research for the community.

The instrument used to obtain the variables of interest was a questionnaire containing 23 items – previously tested with ten parents who were not included in the sample – developed by the researcher, adapted from other studies^(17,18)^, which was divided into three chunks, namely: 1: sociodemographic data (Table 1); 2: description of occurrence of domestic accidents due to children’s falls (Tables 2 and 3); and 3: guidelines on preventive measures for domestic accidents due to children’s falls (Table 4). It is noteworthy that it was not necessary to modify the instrument. The questionnaires were answered individually by each participant, lasting approximately 15 minutes.

**Table 1:** Sociodemographic profile of parents and/or other caregivers of children enrolled in public and private schools (N = 181) – Valença do Piauí, PI, Brazil, 2024.

	N(%)	95%CI^*^	Mean (95%CI)^**^	Med^+^	SD^++^
**Participant**					
Father	7(3.9)	(1.7–7.4)			
Mother	170(93.9)	(89.7–96.7)			
Other caregiver	4(2.2)	(0.8–5.2)			
**Age groups**			31.72(30.64–32.79)	31.00	7.33
18 to 29 years old	81(44.7)	(37.6–52.0)			
30 to 39 years old	71(39.2)	(32.3–46.5)			
40 to 49 years old	28(15.5)	(10.8–21.3)			
50 to 59 years old	1(0.6)	(0.1–2.6)			
**Education**					
Elementary school	20(11.0)	(7.1–16.2)			
High school	110(60.8)	(53.5–67.7)			
Higher education	47(26.0)	(20.0–32.7)			
Illiterate	4(2.2)	(0.8–5.2)			
**Has a graduate degree**					
Specialization	27(14.9)	(10.3–20.6)			
Master’s degree	2(1.1)	(0.2–3.5)			
**Performs other activities besides taking care of children**					
Yes	166(91.7)	(87.0–95.1)			
No	15(8.3)	(4.9–13.0)			
**Employment situation**					
Domestic services (housewife, housewife)	93(51.4)	(44.1–58.6)			
Formal work (signed employment contract)	27(14.9)	(10.3–20.6)			
Informal work	19(10.5)	(6.7–15.6)			
Self-employed	35(19.3)	(14.1–25.6)			
Retired	0(0.0)				
Unemployed	7(3.9)	(1.7–7.4)			
**Family income, considering that the current minimum wage is R$1,412.00**					
Less than one wage	86(47.5)	(40.3–54.8)			
Greater than one wage and less than two wages	51(28.2)	(22.0–35.0)			
Greater than two wages and less than three wages	32(17.7)	(12.7–23.7)			
Greater than three wages and less than four wages	6(3.3)	(1.4–6.7)			
More than four wages	6(3.3)	(1.4–6.7)			
**Marital status**					
Married	82(45.3)	(38.2–52.6)			
Single	57(31.5)	(25.1–38.5)			
Widowed	4(2.2)	(0.8–5.2)			
Divorced/separated	7(3.9)	(1.7–7.4)			
Stable union	31(17.1)	(12.2–23.1)			
**Number of people living in the same house as children**			3.73(3.54–3.93)	4.00	1.36
Up to four people	144(79.5)	(73.2–84.9)			
Between five and nine people	36(19.9)	(14.6–26.2)			
Between ten and 14 people	1(0.6)	(0.1–2.6)			
More than 15 people	0(0.0)				
**Person who stays with children in their absence**					
Grandparent	69(38.0)	(31.3–45.3)			
Sister (brother)	13(7.2)	(4.1–11.6)			
Uncle (aunt)	12(6.6)	(3.7–11.0)			
Other family member	3(1.7)	(0.5–4.4)			
Neighbor	3(1.7)	(0.5–4.4)			
Private caregiver (nanny)	7(3.9)	(1.7–7.4)			
Mother	5(2.8)	(1.1–5.9)			
Father	35(19.3)	(14.1–25.6)			
Takes the child with them	34(18.8)	(13.6–24.9)			

**Caption:** *Confidence Interval for proportion at 95% level;

**Confidence Interval for mean at 95% level;

^+^Med: median;

^++^SD: standard deviation.

**Note:** Decree 11.864, published in the *Diário Oficial da União* on December 27, 2023, which provides for the value of the minimum wage that came into effect on January 1, 2024, being R$ 1,412.00.

**Table 2:** Occurrence of domestic accidents due to children falling – Valença do Piauí, PI, Brazil, 2024.

	N(%)	95%CI^*^
**Child’s current age**		
2 years	31(17.1)	(12.2–23.1)
3 years	41(22.7)	(17.0–29.2)
4 years	55(30.4)	(24.0–37.4)
5 years	54(29.8)	(23.5–36.8)
**Suffered some type of fall at home**		
Yes	156(86.2)	(80.6–153.6)
No	25(13.8)	(9.4–25.4)
Age at which children fell at home**		
< 6 months	19(12.2)	(7.8–18.0)
6 months to 11 months and 29 days	26(16.7)	(11.5–23.1)
1 year	39(25.0)	(18.7–32.2)
2 years	47(30.1)	(23.3–37.6)
3 years	28(17.9)	(12.5–24.5)
4 years	12(7.7)	(4.3–12.7)
5 years	12(7.7)	(4.3–12.7)
**Child’s sex**		
Female	95(52.2)	(44.9–94.4)
Male	86(47.8)	(40.6–86.1)
**Sex of children who fell at home**		
Female	78(50.0)	(42.2–57.8)
Male	78(50.0)	(42.2–57.8)
Rooms in the house where falls occurred**		
Living room	62(39.7)	(32.3–47.6)
Bathroom	22(14.1)	(9.3–20.2)
Balcony	20(12.8)	(8.3–18.7)
Service area	16(10.3)	(6.2–15.7)
Bedroom	91(58.3)	(50.5–65.9)
Kitchen	16(10.3)	(6.2–15.7)
Backyard	32(20.5)	(14.8–27.4)
Outdoor area	18(11.5)	(7.2–17.2)
**Was the child accompanied by an adult at the time of the fall?**		
Yes	133(85.3)	(79.1–90.2)
No	23(14.7)	(9.8–20.9)
**Adult who was with child at the time of the fall**		
Grandparent	16(12.0)	(7.3–16.4)
Sister (brother)	8(6.0)	(2.9–8.0)
Uncle (aunt)	2(1.5)	(0.3–2.7)
Other family member	5(3.8)	(1.4–5.0)
Private caregiver (nanny)	1(0.8)	(0.1–1.5)
Mother	90(67.6)	(59.4–90.2)
Father	11(8.3)	(4.5–11.9)

**Caption:** *Confidence interval for proportion at 95% level;

**Multiple choice variables.

**Table 3:** Type of fall, body parts affected and nature of injuries (N = 156) – Valença do Piauí, PI, Brazil, 2024.

	N(%)	95%CI^*^
Type of fall**		
Bed	88(56.4)	(48.6–64.0)
Hammock	26(16.7)	(11.5–23.1)
Crib	4(2.6)	(0.9–6.0)
Sofa	31(19.9)	(14.2–26.7)
High chair and/or stool	19(12.2)	(7.8–18.0)
Infant stroller	9(5.8)	(2.9–10.3)
Bathtub with changing table	2(1.3)	(0.3–4.0)
Baby walker	4(2.6)	(0.9–6.0)
Other furniture	4(2.6)	(0.9–6.0)
Staircase or steps without handrails	7(4.5)	(2.0–8.6)
Tree	1(0.6)	(0.1–3.0)
Bicycle	31(19.9)	(14.2–26.7)
Slipping on wet floors	41(26.3)	(19.9–33.6)
Slipping on slippery carpets	9(5.8)	(2.9–10.3)
Tripping over toys, furniture, or other items lying around the house	25(16.0)	(10.9–22.4)
Caregiver’s arm	1(0.6)	(0.1–3.0)
Sidewalk	23(14.7)	(9.8–20.9)
Playing with, running away from, or tripping over pets	19(12.2)	(7.8–18.0)
Other	12(7.7)	(4.3–12.7)
**Parts of the body that were hit in the fall** ^**^		
Head	82(52.6)	(44.7–60.3)
Neck	1(0.6)	(0.1–3.0)
Upper limbs	35(22.4)	(16.4–29.4)
Lower limbs	39(25.0)	(18.7–32.2)
Chest	10(6.4)	(3.3–11.1)
Abdomen	6(3.8)	(1.6–7.8)
No part of the body was injured	36(23.1)	(17.0–30.1)
Nature of the injury caused by the fall**		
No injury	94(60.3)	(52.4–67.7)
Contusion	2(1.3)	(0.3–4.0)
Cut and/or laceration	25(16.0)	(10.9–22.4)
Sprain and/or dislocation	8(5.1)	(2.5–9.4)
Fracture	9(5.8)	(2.9–10.3)
Traumatic brain injury	1(0.6)	(0.1–3.0)
Seizure	2(1.3)	(0.3–4.0)
Abrasion/scrape	22(14.1)	(9.3–20.2)
Other	12(7.7)	(4.3–12.7)

**Caption:** *Confidence Interval for proportion at 95% level;

**Multiple choice variables.

**Table 4:** Guidelines on preventive measures for domestic accidents due to children falling (N = 181) – Valença do Piauí, PI, Brazil, 2024.

	N(%)	95%CI^*^
**Have you been advised by a healthcare professional on preventive measures for child falls at home**		
Yes	62(34.3)	(27.6–62.4)
No	119(65.7)	(58.6–119.4)
**If the answer to the previous question is “yes”, the professional who provided guidance**		
Nursing assistant and/or technician	7(11.3)	(5.2–7.9)
Community health worker	15(24.2)	(14.9–15.9)
Nurse	13(21.0)	(12.3–13.3)
Doctor	12(19.3)	(11.0–12.5)
Other healthcare professional	15(24.2)	(14.9–15.9)

**Caption:** ^*^Confidence Interval for proportion at the 95% level.

Chunk 1 variables were study participant, age, education, whether they have a graduate degree, whether they have another activity in addition to caring for the child, employment status, family income, marital status, number of people living in the same house as the child and person who stays with the child in the absence of the main caregiver.

Subsequently, chunk 2 variables were child’s current age, child’s age when they fell at home, the occurrence of a child fall at home, child’s sex and whether they fell at home, type of fall, the body parts affected by the fall, the nature of the injury caused by the fall, the room(s) in the house where the fall(s) occurred, the time of the fall, whether the child was accompanied by an adult in the room(s) of the house where they fell, and which adult was with the child at the time of the fall.

Finally, in chunk 3, the following variables were investigated: have you ever been advised by a healthcare professional on preventive measures for domestic accidents caused by children falling? If yes to the previous question, which professional provided guidance on preventive measures for domestic accidents caused by children falling?

Although the researcher instructed participants to answer the questionnaire carefully and clarified any doubts, the findings are susceptible to information bias due to data that may have been omitted, especially due to forgetting all episodes of falls, due to the time elapsed between the occurrence of the accidents and participation in the study.

### Data Analysis and Processing

The collected data were organized and entered into Microsoft Office Excel 2016^®^. For inferential statistical analysis, the Statistical Package for the Social Sciences (SPSS) version 26 was used.

A descriptive analysis was performed based on absolute and relative frequencies of qualitative variables. Moreover, position (mean and median) and dispersion (standard deviation) measures were used for quantitative variables, and the 95% Confidence Interval was calculated when applicable.

### Ethical Aspects

This study was approved by the *Universidade Federal do Piauí* (UFPI) Research Ethics Committee (REC), under Opinion 6.516.150. Parents and/or other caregivers who agreed to participate in the study signed the Informed Consent Form (ICF) prior to completing the data collection instrument. In addition to this, the other ethical recommendations of Resolution 466/2012 of the Brazilian National Health Council were met^(19)^. It should be added that the study received institutional authorization from the Municipal Department of Education of Valença do Piauí.

## RESULTS

The study included 181 parents and/or other caregivers of children enrolled in public and private schools in Valença do Piauí, Piauí. The majority of the sample was composed of mothers (93.9%), aged between 18 and 29 years (44.7%), with high school education (60.8%) and married (45.3%). There was a greater number of participants who performed other activities in addition to caring for children (91.7%), especially domestic services (51.4%), with a family income of less than one wage (47.5%). Most participants had up to four people living in the same house (79.5%) and, in the absence of the main caregiver, the child stayed with their grandparents (38%) (Table 1).

The majority of the 181 children were currently 4 years old (30.4%), were female (52.2%), and 86.2% suffered some type of fall at home. Regarding the age at which children suffered falls, 2 years of age predominated (30.1%), and the bedroom (58.3%) was the room where these accidents occurred most, although they were accompanied by an adult (85.3%), mainly their mother (67.6%) (Table 2).

Regarding the type of fall, the most common types were falls from bed (56.4%), slipping on a wet floor (26.3%), sofa (19.9%), bicycle (19.9%) and hammock (16.7%), with the most affected body parts being the head (52.6%), lower limbs (25%) and upper limbs (22.4%). Most falls did not cause injuries (60.3%). There was a predominance of cuts and/or lacerations (16%), abrasions/scrapes (14.1%) and fractures (5.8%) caused by falls (Table 3).

The majority of participants (65.7%) reported that they had not received guidance on preventive measures for domestic accidents due to children falling. In cases where study participants received guidance from healthcare professionals (34.3%), they mainly reported the community health worker (CHW) (24.2%), another professional (24.2%) and the nurse (21%) (Table 4).

## DISCUSSION

In this research, it was found that most caregivers were mothers, young adults, with high school education and housewives, supporting other studies^(11,17,18)^. In fact, female care stands out over male care, as there is still little personal involvement of fathers with children, although their participation in care contributes to protecting infants from domestic accidents^(13)^.

A Brazilian study carried out in Rio Grande do Sul, with men, revealed that healthcare services still need to implement strategies for including fathers and encourage their participation in educational activities, as they felt excluded from the care provided by professionals, showing that health intervention strategies need to be (re)constructed, in order to implement actions that include fathers as an active participant in care^(20)^.

In this study, the occurrence of child falls at home was high, as identified in another study^(11)^, indicating the need to adopt active and continuous supervision by adults and prioritize interventions aimed at reducing child exposure to such injuries, in order to prevent unintentional injuries in the domestic context^(21)^.

At a global level, the occurrence of domestic accidents in childhood demands investment in public policies that reduce social inequities and exposure to risks, paying attention to the different contexts in which children are inserted^(22)^. Therefore, it is considered necessary to implement preventive measures that involve the participation of the entire society, such as families, healthcare and education professionals, governments, religious institutions, among other groups.

In relation to the profile of children and the occurrence of accidents, in this investigation, it was observed that there was a higher frequency of falls at the age of 2 years, followed by 1 year, while ages 4 and 5 years were less affected. The literature shows that the child’s age can influence the occurrence of domestic accidents. Thus, supporting this finding, an integrative review study revealed that, generally, accidents are less frequent among children under 1 year of age and that the peak occurrence of domestic accidents occurred with children between 1 and 2 years of age, reducing to 4 years^(22)^.

Furthermore, the same number of falls were identified for boys and girls. However, national and international studies show that males are more likely to have domestic accidents^(18,22)^, which can be attributed to the fact that boys are more exposed to risky situations, such as riding bicycles, playing with balls, riding bicycles, rollerblading, among others^(23)^.

It is understood that direct supervision by an adult constitutes one of the protective factors to prevent accidents from occurring in childhood, since more attentive care for a vulnerable person allows for the early identification of risks^(13)^. In this investigation, however, it was found that most children who fell at home were accompanied by an adult, a finding that supports another Brazilian study^(24)^, which highlights the lack of close supervision and the family’s unpreparedness to identify characteristics and behaviors that are specific to child development and that lead to accidents. This fact may also be related to the lack of help with household chores and childcare, which are usually carried out by a single person^(25)^.

A study conducted with caregivers of children under 5 years of age revealed that domestic accidents are the result of a set of factors, such as adult caregivers’ carelessness and recognition (or lack thereof) of children’s development process. Thus, it is understood that the causes of such accidents are complex, as they involve the caregiver, the child, the family and the environment^(13)^. Hence, it is necessary to understand the child’s growth and development, in order to maintain a safe environment according to each phase^(3)^.

In this study, the most common factors were falling from bed, slipping on wet floors, sofas, bicycles and hammocks. Another study conducted in Piauí found that high hammocks, the presence of stairs or steps without handrails and exits and passages filled with toys, furniture, boxes or other items that could be obstructive were associated with the risk of falls in children under 5 years of age^(17)^.

International research has identified that several modifiable factors were associated with a greater chance of children between the ages of 0 and 4 falling from furniture, namely: not using safety barriers in the rooms of the house; leaving children on elevated surfaces; changing diapers in high places; not teaching children safety rules; and climbing or playing on furniture^(26)^.

Thus, a study carried out in New Zealand found that low-cost modifications and repairs in the home can reduce injuries, such as handrails for steps, stairs and bathrooms, repairs to window locks, adequate lighting, non-slip bath mats, among others^(27)^.

Furthermore, it was found that the part of the body most affected during a fall was the head/face (52.6%), supporting other studies^(11,24,26)^, while in other studies there was a predominance of lower limbs^(17)^ and upper limbs^(18)^. As for the nature of the injuries caused by the fall, there was a predominance of cuts and/or lacerations, abrasions/scratches and fractures, converging with the results of a multicenter study developed in the United Kingdom with children aged 0 to 4 years who suffered falls from furniture^(26)^.

Hence, research carried out in Australia, with children under 3 years old, showed that falls were the main cause of traumatic brain injury and head injuries, which occurred through different mechanisms, such as sofas, beds, baby carriages, tables, high chairs, shopping carts, diaper changing tables, bunk beds, caregiver’s arms, stairs, furniture, among others^(28)^.

Another study showed that 52.8% of children up to 6 months of age are placed in their parents’ bed for nighttime sleep, even in the presence of a crib^(29)^, which poses a risk of falls. Thus, research carried out in Mato Grosso revealed that, of the 113 children studied, 31% suffered some type of accident up to 6 months of age, with a higher occurrence of falls (80%), mainly from the bed (45.7%)^(24)^.

Regarding guidelines on preventive measures for domestic accidents caused by children falling, most participants reported that they were not given any guidance, a finding that coincided with another study^(18)^. In this context, research carried out in Rio de Janeiro with caregivers revealed a lack of knowledge of information about accident prevention contained in the Child Health Card, which is a resource for monitoring child development^(25)^, highlighting gaps in knowledge on the subject.

Among the participants who received guidance, the majority reported that they were guided by CHW and other professionals, followed by nurses, in accordance with another study^(18)^. Thus, continuing health education practices are essential to qualify CHW, in order to work effectively on children’s health^(30)^.

In the health education scenario, nursing stands out, as it has a holistic vision and critical-reflective reasoning, being responsible for planning, implementing and assessing educational actions based on the realization of a situational diagnosis of the target audience^(31)^. Therefore, one of the nurses’ duties is to guide and encourage the adoption of child protection measures^(3)^ during childcare consultations and/or in healthcare waiting rooms^(25)^, in schools, churches, shelters, sports and leisure areas, among others.

From this perspective, among the objectives and goals of the Brazilian National Plan for Early Childhood for accident prevention in early childhood, in the health area, are guidance and awareness of parents and guardians of children from the beginning of pregnancy, using television campaigns, leaflets, safety checklists, posters, meetings in health centers, early childhood education establishments and schools, and home visits by Family Health teams^(32)^.

Furthermore, through the Brazilian National Policy for Reducing Morbidity and Mortality from Accidents and Violence, the Ministry of Health defined guidelines such as: promoting the adoption of safe and healthy behaviors and environments; monitoring the occurrence of accidents and violence; training human resources; and supporting the development of studies and research^(5)^.

The notoriety of the results of this research confirms the need to invest efforts in well-planned educational interventions aimed at children’s caregivers, because it is essential to raise awareness among families about the imminent responsibility of protecting them from domestic accidents, protecting them from injuries that can be avoided through changes in behavior and the adoption of a proactive stance, by intervening in anticipation of risk factors, transforming the home into a safe space.

Thus, by adding knowledge about the occurrence of domestic accidents due to children falling, it is expected to provide support for developing, validating and implementing educational technologies that aim to guide families on the adoption of safe practices, in order to prevent such accidents, as well as carrying out continuous strategic actions aimed at training and qualifying healthcare professionals, especially those who accompany children in childcare consultations.

Among the limitations of this study, it is worth noting that the instrument used scored only objective variables, not allowing the identification of the entire context of an accident, the repercussions, perceptions and reactions of parents and/or other caregivers in the face of a fall suffered by a child. Furthermore, the absence of a professional sign language interpreter made it impossible for two deaf couples to participate.

As this is a cross-sectional study, carried out in a specific and punctual context, in the countryside of the northeastern state, it is not feasible to generalize the interpretations to other regions, considering the peculiarities of each location.

## CONCLUSION

Mothers are primarily responsible for caring for children as well as carrying out household chores and other tasks. There was a high incidence of falls in children at home, for both girls and boys, especially in the 1 to 2 year old age group. Furthermore, most falls occurred in the bedroom, living room and backyard, mainly from the bed, slipping on wet floors, from the sofa, from the bicycle, from the hammock, tripping over toys and from the sidewalk, which caused injuries such as cuts, abrasions, fractures, sprains, bruises, convulsions, traumatic brain injuries and others, affecting, in most cases, the head/face, lower and upper limbs.

It is recommended that healthcare professionals work in interdisciplinary teams in order to guide families on preventive measures for occurrence of domestic accidents due to children falling, occupying different social spaces, such as schools, churches, healthcare services, sports and leisure areas as well as applying different strategies to reach the target audience, whether through group activities in the community or through individual activities, mainly in the home where children live.

Despite being a widely discussed topic worldwide, it is suggested that studies be carried out aimed at the development, validation and implementation of alternative educational resources that are available to the entire typical and disabled community, including in a playful and interactive manner, for both adults and children and young people, such as printed materials, such as booklets, folders as well as applications, games, models, music, audiobooks and videos, with a view to ensuring the population’s accessibility to strategies for preventing domestic accidents due to children’s falls.
