# Supplementary material for: Domestic accidents due to children’s falls: a cross-sectional study
Source: Rev Esc Enferm USP. 2024 Oct 21;58:e20240192. doi: 10.1590/1980-220X-REEUSP-2024-0192en (PMC11499244; doi:10.1590/1980-220X-REEUSP-2024-0192en)
Supplement: Translation

# Translation: Acidentes domésticos por queda infantil: estudo transversal

**DOI:** 10.1590/1980-220X-REEUSP-2024-0192pt

**Authors:** Abiúde Nadabe e Silva, Lídya Tolstenko Nogueira, Ana Roberta Vilarouca da Silva

**Affiliations:** 1 Universidade Federal do Piauí, Programa de Pós-Graduação em Enfermagem, Teresina, PI, Brasil.

**Keywords:** Acidentes por Quedas, Acidentes Domésticos, Pré-Escolar, Enfermagem

## Abstract

**Objetivo:**

Descrever a ocorrência de acidentes domésticos por queda infantil.

**Método:**

Trata-se de estudo descritivo, transversal, realizado com 181 pais e/ou outros cuidadores de crianças com idade entre 2 e 5 anos. Os dados foram coletados nos meses de março e abril de 2024, por meio de um questionário que contém 23 itens, os quais foram submetidos à análise descritiva.

**Resultados:**

A amostra foi composta em sua maioria por mães (93,9%), com predomínio de crianças do sexo feminino (52,2%), sendo que 86,2% dos infantes sofreram algum tipo de queda no domicílio. Predominaram quedas da cama (56,4%), atingindo a cabeça/face (52,6%), ocasionando, principalmente, corte/laceração (16%).

**Conclusão:**

Os resultados evidenciam que é imprescindível sensibilizar as famílias quanto à iminente responsabilidade de proteger as crianças dos acidentes domésticos, resguardando-as de lesões que podem ser evitadas por meio de mudanças de comportamentos e adoção de uma postura proativa ao intervir de forma antecipatória nos fatores de risco.

## INTRODUÇÃO

A infância caracteriza-se por diferentes etapas do desenvolvimento, destacando-se a primeira infância – crianças até 6 anos de idade. É durante essa fase que a maioria das habilidades se desenvolvem, como falar, andar, correr, pular e a capacidade para aprender^(1)^. Por conseguinte, à medida que a criança cresce, sua curiosidade é despertada e se torna algo constante no cotidiano, o que aumenta o risco de sofrer acidentes^(2)^.

Dessa maneira, os acidentes têm contribuído para aumentar a taxa de morbimortalidade infantil, já que estão intimamente relacionados à própria fase da infância, além de refletir, muitas vezes, a carência de capacidade protetora da família e o desconhecimento das situações de risco que permeiam o cotidiano da criança^(3)^.

Nesse sentido, os trabalhadores da Atenção Primária à Saúde, por atuarem nas comunidades e manterem contato mais próximo com as famílias, podem desenvolver ações educativas para prevenir acidentes, principalmente durante as visitas domiciliares. Então, faz-se necessário avaliar os aspectos de segurança no ambiente doméstico, orientar os responsáveis acerca de medidas simples para a segurança da criança, realização de campanhas educativas locais e capacitação dos profissionais de saúde, considerando-se os fatores de risco, as vulnerabilidades e o estágio de desenvolvimento infantil^(4)^.

Acidente é definido como o evento não intencional, mas evitável, que pode ocorrer no domicílio, trabalho, trânsito, escola, áreas de esportes e de lazer, e ocasionar lesões físicas e/ou emocionais^(5)^. Os acidentes são provocados por vários fatores previsíveis, combinados às condições físicas e psicológicas das pessoas, bem como à estrutura presente no meio ambiente físico, social, cultural e organizacional^(6)^. Queda, por sua vez, consiste no evento em que a pessoa, inadvertidamente, cai no solo, piso ou em outro nível abaixo daquele em que se encontrava^(7).^

Assim, compreende-se que há vários fatores desencadeantes de acidentes, como condições de habitação, nível socioeconômico, condições de segurança do ambiente externo, ausência de locais adequados para a recreação, falta de informações sobre a prevenção dos acidentes, características psicológicas e físicas da criança, entre outros^(3)^. Além de acarretar custos sociais, econômicos e emocionais, os acidentes também causam sequelas que repercutem na família e na sociedade, afligindo muitas crianças^(8)^.

Os acidentes por quedas envolvendo as crianças acontecem dentro de casa e nas suas imediações, nas escolas, nos parques infantis, durante atividades esportivas e de lazer, e ao ar livre^(7)^. No período de 2000 a 2019, a nível mundial, as quedas foram responsáveis por cerca de 31.818 mortes de crianças e adolescentes menores de 15 anos^(9)^.

Dados do Viva Inquérito a respeito das características gerais do atendimento por queda acidental em serviços sentinelas de urgência e emergência, em 20 capitais de estados brasileiros e no Distrito Federal, revelaram que 57,3% das quedas ocorreram na residência da vítima, sendo queda no mesmo nível (57,2%), escada ou degrau (18,3%), leito, berço, rede ou outra mobília (10,4%), andaime, árvore, telhado ou laje (4,8%), entre outros^(10)^.

As quedas acidentais causaram lesões como contusão, entorse ou luxação (59,3%), trauma (20,2%) e corte ou laceração (14,2%), atingindo cabeça ou pescoço (23,4%), tronco (14,3%), membros superiores (28,5%) e inferiores (33,8%), sendo que 9,1% precisaram de internação hospitalar. Os atendimentos por queda acidental na residência foram mais frequentes entre as faixas extremas de idade de 0 a 9 anos (71,6%) e 60 e mais anos (71,8%)^(10)^.

Estudo realizado no Ceará revelou que a maioria dos acidentes domésticos envolvendo crianças da primeira infância aconteceu em populações de baixa renda, escolaridade média, mães jovens e com várias pessoas morando na mesma casa, sendo que a queda foi o tipo de acidente mais prevalente (66,6%)^(11)^. Outra pesquisa desenvolvida no Brasil evidenciou que a maioria das quedas envolvendo crianças ocorreu em casa (60,7%), sendo que 52,3% dos acidentes aconteceram com crianças de 0 a 5 anos^(12)^.

Nesse contexto, a enfermagem tem responsabilidade importante nos aspectos que envolvem a saúde das crianças, sobretudo na prevenção de acidentes, posto que os cuidadores necessitam ser sensibilizados e orientados quanto aos riscos ambientais, no intuito de promover mudanças de comportamento na forma de cuidar e evitar episódios de queda^(13)^.

Embora as literaturas nacional e internacional apresentem informações acerca do perfil de acidentes domésticos por quedas na infância, entende-se que cada região tem suas próprias nuances, por isso este estudo se faz necessário, no sentido de apresentar um diagnóstico situacional do local de interesse para, a partir de então, projetar medidas de prevenção conforme as necessidades evidenciadas. Ademais, após busca manual de artigos publicados nos últimos cinco anos, em alguns periódicos brasileiros, observou-se que o escopo de publicações acerca desta temática é restrito.

Por conseguinte, a partir da identificação dos principais tipos de queda, este estudo fornece conhecimento que pode direcionar o desenvolvimento de programas e materiais educativos, a fim de instruir a família quanto à prevenção de queda infantil no domicílio, intervindo, sobretudo, nas situações de risco modificáveis. Assim, o presente trabalho objetivou descrever a ocorrência de acidentes domésticos por queda infantil.

## MÉTODO

### Desenho do Estudo

Trata-se de estudo descritivo, transversal. A comunicação dos dados seguiu as recomendações da declaração *STrengthening the Reporting of OBservational studies in Epidemiology* (STROBE)^(14)^. Esta pesquisa deriva de estudo multimétodos intitulado “Evidências de validade de cartilha educativa para a prevenção de acidentes domésticos por queda infantil”.

### Local

O estudo foi realizado em Valença do Piauí, Piauí, localizada a 210 km da capital, Teresina, com área territorial de 1.333,722 km^2^, 22.279 habitantes e Índice de Desenvolvimento Humano (IDH) de 0,647, sendo que o salário médio mensal dos trabalhadores formais é de 1,6 salários mínimos^(15)^.

Nesse local, há quatro escolas públicas municipais e três escolas privadas, as quais trabalham com crianças entre 2 e 5 anos de idade. Dessa forma, fez-se um sorteio pelo qual selecionaram-se duas escolas públicas e uma escola privada. Optou-se por realizar a pesquisa apenas na zona urbana, em decorrência da distância geográfica e dificuldade de acesso às comunidades da zona rural.

### População, Critérios de Inclusão e Exclusão

A população do estudo foi composta por pais e/ou outros cuidadores de 340 crianças, sendo 138 da escola A, 117 da escola B e 85 da escola C. Ressalta-se que, para cada criança, foi pesquisado apenas um cuidador, seja o pai, a mãe ou outro responsável.

Os critérios de elegibilidade para estabelecer os participantes do estudo foram ser pai, mãe ou outro cuidador de crianças entre 2 e 5 anos de idade, e ser maior de idade, isto é, ter idade igual ou superior a 18 anos. Os critérios de exclusão foram ser pai, mãe ou outro cuidador com quaisquer dificuldades cognitivas, comportamentais, comprometimento visual e/ou auditivo severos que inviabilizassem a leitura, a compreensão e o preenchimento do questionário, bem como cuidadores de crianças que possuíssem incapacidades motoras graves.

Por conseguinte, dois casais surdos – mães e pais – foram excluídos do estudo pela ausência de um profissional intérprete de libras que pudesse transmitir as informações de modo compreensível.

Ressalta-se que quatro cuidadoras, autodeclaradas analfabetas, manifestaram interesse em participar da pesquisa. Nesse contexto, compreendeu-se que, embora não soubessem ler nem escrever, estavam aptas para ouvir as perguntas e expressar suas vivências, verbalmente. Com esse entendimento, a pesquisadora fez a leitura do questionário, em voz audível, para cada participante analfabeta, individualmente, e o preencheu de acordo com as respostas relatadas oralmente, sendo, portanto, incluídas na amostra.

Tais relatos do diário de campo são relevantes, porquanto revelam as diferentes necessidades da população e despertam os pesquisadores para buscar recursos educativos e instrumentos de pesquisa alternativos, no sentido de realizar atividades de promoção da saúde e segurança que sejam acessíveis a todos.

### Cálculo da Amostra

Para calcular a amostra, utilizou-se a fórmula para população finita, estratificada por proporção, com um erro amostral de 5% e um nível de confiança de 95%^(16)^, que resultou em uma amostra mínima de 180,61 ≅ 181 participantes.

### Coleta de Dados

Os dados foram coletados pela pesquisadora principal, nos meses de março e abril de 2024, após contato prévio com as diretoras de cada escola, as quais procederam com presteza e concordaram que a coleta de dados fosse realizada no momento em que os pais e/ou outros cuidadores estivessem deixando as crianças no ambiente escolar. Dessa forma, a pesquisadora reuniu os participantes do estudo em grupos de 30 pessoas, em sala reservada nas escolas, nos turnos matutino e vespertino, em dias alternados, até alcançar o quantitativo da amostra.

Ressalta-se que a direção de cada escola informou com antecedência – via mensagem nos grupos de *WhatsApp* ^®^ de cada turma – aos pais e/ou cuidadores que, nos dias seguintes, a enfermeira pesquisadora estaria na escola para realizar a pesquisa. Mesmo assim, em ambas as instituições educacionais, muitas pessoas se recusaram a responder ao questionário, alegando falta de tempo e/ou o não desejo de participar do estudo, apesar de a pesquisadora ter explicado, presencialmente, os objetivos e benefícios da pesquisa para a comunidade.

O instrumento utilizado para obter as variáveis de interesse foi um questionário que contém 23 itens – previamente testado com dez pais que não foram incluídos na amostra – elaborado pela pesquisadora, adaptado de outros estudos^(17,18)^, o qual foi dividido em três blocos, a saber: 1: dados sociodemográficos (Tabela 1); 2: descrição da ocorrência de acidentes domésticos por queda infantil (Tabelas 2 e 3); e 3: orientações sobre medidas de prevenção para acidentes domésticos por queda infantil (Tabela 4). Destaca-se que não foi necessário modificar o instrumento. Os questionários foram respondidos, individualmente, por cada participante, com duração de, aproximadamente, 15 minutos.

**Tabela 1:** Perfil sociodemográfico de pais e/ou outros cuidadores de crianças matriculadas em escolas da rede pública e privada (N = 181) – Valença do Piauí, PI, Brasil, 2024.

	N(%)	IC95%^*^	Média (IC95%)^**^	Med^+^	DP^++^
**Participante**					
Pai	7(3,9)	(1,7–7,4)			
Mãe	170(93,9)	(89,7–96,7)			
Outro cuidador	4(2,2)	(0,8–5,2)			
**Grupos de idade**			31,72(30,64–32,79)	31,00	7,33
18 a 29 anos	81(44,7)	(37,6–52,0)			
30 a 39 anos	71(39,2)	(32,3–46,5)			
40 a 49 anos	28(15,5)	(10,8–21,3)			
50 a 59 anos	1(0,6)	(0,1–2,6)			
**Escolaridade**					
Ensino Fundamental	20(11,0)	(7,1–16,2)			
Ensino **M**édio	110(60,8)	(53,5–67,7)			
Ensino Superior	47(26,0)	(20,0–32,7)			
Analfabeto	4(2,2)	(0,8–5,2)			
**Possui pós–graduação**					
Especialização	27(14,9)	(10,3–20,6)			
Mestrado	2(1,1)	(0,2–3,5)			
**Exerce outra atividade além de cuidar da criança**					
Sim	166(91,7)	(87,0–95,1)			
Não	15(8,3)	(4,9–13,0)			
**Situação laboral**					
Serviços domésticos (dona de casa, do lar)	93(51,4)	(44,1–58,6)			
Trabalho formal (carteira assinada)	27(14,9)	(10,3–20,6)			
Trabalho informal	19(10,5)	(6,7–15,6)			
Autônomo(a)	35(19,3)	(14,1–25,6)			
Aposentado(a)	0(0,0)				
Desempregado(a)	7(3,9)	(1,7–7,4)			
**Renda familiar, considerando–se que o valor do salário mínimo atual é R$ 1.412,00**					
menor que um salário	86(47,5)	(40,3–54,8)			
maior que um salário e menor que dois salários	51(28,2)	(22,0–35,0)			
maior que dois salários e menor que três salários	32(17,7)	(12,7–23,7)			
maior que três salários e menor que quatro salários	6(3,3)	(1,4–6,7)			
mais de quatro salários	6(3,3)	(1,4–6,7)			
**Estado civil**					
casado(a)	82(45,3)	(38,2–52,6)			
solteiro(a)	57(31,5)	(25,1–38,5)			
viúvo(a)	4(2,2)	(0,8–5,2)			
divorciado(a)/desquitado(a)	7(3,9)	(1,7–7,4)			
união estável	31(17,1)	(12,2–23,1)			
**Quantidade de pessoas que residem na mesma casa que a criança**			3,73(3,54–3,93)	4,00	1,36
Até quatro pessoas	144(79,5)	(73,2–84,9)			
Entre cinco e nove pessoas	36(19,9)	(14,6–26,2)			
Entre dez e 14 pessoas	1(0,6)	(0,1–2,6)			
Mais de 15 pessoas	0(0,0)				
**Pessoa que fica com a criança na sua ausência**					
Avó(ô)	69(38,0)	(31,3–45,3)			
Irmã(ão)	13(7,2)	(4,1–11,6)			
Tio(a)	12(6,6)	(3,7–11,0)			
Outro familiar	3(1,7)	(0,5–4,4)			
Vizinho(a)	3(1,7)	(0,5–4,4)			
Cuidador particular (babá)	7(3,9)	(1,7–7,4)			
Mãe	5(2,8)	(1,1–5,9)			
Pai	35(19,3)	(14,1–25,6)			
Leva a criança	34(18,8)	(13,6–24,9)			

**Legenda:** ^*^Intervalo de Confiança para proporção ao nível de 95%;

^**^Intervalo de Confiança para média ao nível de 95%;

^+^Med: mediana;

^++^DP: desvio padrão.

**Nota:** Decreto nº 11.864, publicado no Diário Oficial da União, em 27 de dezembro de 2023, que dispõe sobre o valor do salário mínimo que passou a vigorar a partir de 1º de janeiro de 2024, sendo R$ 1.412,00.

**Tabela 2:** Ocorrência de acidentes domésticos por queda infantil – Valença do Piauí, PI, Brasil, 2024.

	N(%)	IC95%^*^
**Idade atual da criança**	
2 anos	31(17,1)	(12,2–23,1)
3 anos	41(22,7)	(17,0–29,2)
4 anos	55(30,4)	(24,0–37,4)
5 anos	54(29,8)	(23,5–36,8)
**Sofreu algum tipo de queda em casa**	
Sim	156(86,2)	(80,6–153,6)
Não	25(13,8)	(9,4–25,4)
**Idade que a criança sofreu queda em casa** ^**^	
< 6 meses	19(12,2)	(7,8–18,0)
6 meses a 11 meses e 29 dias	26(16,7)	(11,5–23,1)
1 ano	39(25,0)	(18,7–32,2)
2 anos	47(30,1)	(23,3–37,6)
3 anos	28(17,9)	(12,5–24,5)
4 anos	12(7,7)	(4,3–12,7)
5 anos	12(7,7)	(4,3–12,7)
**Sexo da criança**	
Feminino	95(52,2)	(44,9–94,4)
Masculino	86(47,8)	(40,6–86,1)
**Sexo da criança que sofreu queda em casa**			
Feminino	78(50,0)	(42,2–57,8)
Masculino	78(50,0)	(42,2–57,8)
**Cômodos da casa onde ocorreram as quedas** ^**^	
Sala	62(39,7)	(32,3–47,6)
Banheiro	22(14,1)	(9,3–20,2)
Varanda	20(12,8)	(8,3–18,7)
Área de serviço	16(10,3)	(6,2–15,7)
Quarto	91(58,3)	(50,5–65,9)
Cozinha	16(10,3)	(6,2–15,7)
Quintal	32(20,5)	(14,8–27,4)
Área externa	18(11,5)	(7,2–17,2)
**A criança estava acompanhada de algum adulto no momento da queda?**	
Sim	133(85,3)	(79,1–90,2)
Não	23(14,7)	(9,8–20,9)
**Adulto que estava com a criança no momento da queda**	
Avó(ô)	16(12,0)	(7,3–16,4)
Irmã(ão)	8(6,0)	(2,9–8,0)
Tio(a)	2(1,5)	(0,3–2,7)
Outro familiar	5(3,8)	(1,4–5,0)
Cuidador particular (babá)	1(0,8)	(0,1–1,5)
Mãe	90(67,6)	(59,4–90,2)
Pai	11(8,3)	(4,5–11,9)

**Legenda:** ^*^Intervalo de Confiança para proporção ao nível de 95%;

^**^Variáveis de múltipla escolha.

**Tabela 3:** Tipo de queda, partes do corpo atingidas e natureza das lesões (N = 156) – Valença do Piauí, PI, Brasil, 2024.

	N(%)	IC95%^*^
**Tipo de queda** ^**^	
Cama	88(56,4)	(48,6–64,0)
Rede	26(16,7)	(11,5–23,1)
Berço	4(2,6)	(0,9–6,0)
Sofá	31(19,9)	(14,2–26,7)
Cadeira alta e/ou banqueta	19(12,2)	(7,8–18,0)
Carrinho infantile	9(5,8)	(2,9–10,3)
Banheira com trocador de fraldas	2(1,3)	(0,3–4,0)
Andador infantile	4(2,6)	(0,9–6,0)
Outra mobília	4(2,6)	(0,9–6,0)
Escada ou degraus sem corrimão	7(4,5)	(2,0–8,6)
Árvore	1(0,6)	(0,1–3,0)
Bicicleta	31(19,9)	(14,2–26,7)
Escorregão em piso molhado	41(26,3)	(19,9–33,6)
Escorregão em tapete escorregadio	9(5,8)	(2,9–10,3)
Tropeção em brinquedos, móveis ou outros itens espalhados pela casa	25(16,0)	(10,9–22,4)
Braço do cuidador	1(0,6)	(0,1–3,0)
Calçada	23(14,7)	(9,8–20,9)
Ao brincar, fugir ou tropeçar em animais de estimação	19(12,2)	(7,8–18,0)
Outros	12(7,7)	(4,3–12,7)
**Partes do corpo que foram atingidas na queda** ^**^	
Cabeça	82(52,6)	(44,7–60,3)
Pescoço	1(0,6)	(0,1–3,0)
Membros superior**e**s	35(22,4)	(16,4–29,4)
Membros inferior**e**s	39(25,0)	(18,7–32,2)
Tórax	10(6,4)	(3,3–11,1)
Abdômen	6(3,8)	(1,6–7,8)
Nenhuma parte do corpo foi atingida	36(23,1)	(17,0–30,1)
**Natureza da lesão ocasionada pela queda** ^**^	
Sem lesão	94(60,3)	(52,4–67,7)
Contusão	2(1,3)	(0,3–4,0)
Corte e/ou laceração	25(16,0)	(10,9–22,4)
Entorse e/ou luxação	8(5,1)	(2,5–9,4)
Fratura	9(5,8)	(2,9–10,3)
Traumatismo cranioencefálico	1(0,6)	(0,1–3,0)
Convulsão	2(1,3)	(0,3–4,0)
Escoriação/raladura	22(14,1)	(9,3–20,2)
Outros	12(7,7)	(4,3–12,7)

**Legenda:** ^*^Intervalo de Confiança para proporção ao nível de 95%;

^**^Variáveis de múltipla escolha.

**Tabela 4:** Orientações sobre medidas de prevenção para acidentes domésticos por queda infantil (N = 181) – Valença do Piauí, PI, Brasil, 2024.

	N(%)	IC95%^*^
**Foi orientado(a) por algum profissional da área da saúde sobre medidas de prevenção para a queda infantil em casa**	
Sim	62(34,3)	(27,6–62,4)
Não	119(65,7)	(58,6–119,4)
**Em caso de “sim” na pergunta anterior, profissional que orientou**	
Auxiliar e/ou técnico(a) de enfermagem	7(11,3)	(5,2–7,9)
Agente comunitário de saúde	15(24,2)	(14,9–15,9)
Enfermeiro(a)	13(21,0)	(12,3–13,3)
Médico(a)	12(19,3)	(11,0–12,5)
Outro profissional de saúde	15(24,2)	(14,9–15,9)

**Legenda:** ^*^Intervalo de Confiança para proporção ao nível de 95%.

As variáveis do bloco 1 foram participante do estudo, idade, escolaridade, se possui pós-graduação, se exerce outra atividade além de cuidar da criança, situação laboral, renda familiar, estado civil, quantidade de pessoas que residem na mesma casa que a criança e pessoa que fica com a criança na ausência do cuidador principal.

Na sequência, as variáveis do bloco 2 foram idade atual da criança, idade que a criança tinha quando sofreu queda em casa, ocorrência de queda infantil em casa, sexo da criança e se sofreu queda em casa, tipo de queda, partes do corpo atingidas na queda, natureza da lesão ocasionada pela queda, em qual(is) ambiente(s) da casa ocorreu(ram) a(s) queda(s), no momento da queda, se a criança estava acompanhada de algum adulto no ambiente da casa onde caiu e qual era o adulto que estava com a criança no momento da queda.

Por fim, no bloco 3, foram investigadas as seguintes variáveis: você já foi orientado por algum profissional da área da saúde sobre medidas de prevenção para acidentes domésticos por queda infantil? E em caso de sim na questão anterior, qual foi o profissional que orientou sobre medidas de prevenção para acidentes domésticos por queda infantil?

Embora a pesquisadora tenha orientado os participantes a responderem ao questionário com atenção e sanado as dúvidas, os achados estão susceptíveis ao viés de informação por dados que podem ter sido omitidos, sobretudo por esquecimento de todos os episódios de queda, devido ao tempo decorrido entre a ocorrência dos acidentes e a participação no estudo.

### Análise e Tratamento dos Dados

Os dados coletados foram organizados e digitados no editor Microsoft Office Excel 2016^®^. Para a análise estatística inferencial, utilizou-se o software Statistical Package for the Social Sciences (SPSS), versão 26.

Realizou-se análise descritiva a partir das frequências absolutas e relativas das variáveis qualitativas. Além disso, utilizaram-se as medidas de posição (média e mediana) e de dispersão (desvio padrão) para as variáveis quantitativas, sendo que o Intervalo de Confiança de 95% foi calculado quando pertinente.

### Aspectos Éticos

Este estudo foi aprovado pelo Comitê de Ética em Pesquisa (CEP) da Universidade Federal do Piauí (UFPI), sob Parecer nº 6.516.150. Os pais e/ou outros cuidadores que aceitaram participar do estudo assinaram o Termo de Consentimento Livre e Esclarecido (TCLE) previamente ao preenchimento do instrumento para coleta de dados. Além disso, foram atendidas as demais recomendações éticas da Resolução nº 466/2012 do Conselho Nacional de Saúde^(19)^. Acrescenta-se que o estudo recebeu a autorização institucional da Secretaria Municipal de Educação de Valença do Piauí.

## RESULTADOS

Participaram desta pesquisa 181 pais e/ou outros cuidadores de crianças matriculadas em escolas da rede pública e privada de Valença do Piauí, Piauí, sendo que a maior parte da amostra foi composta por mães (93,9%), com idade entre 18 e 29 anos (44,7%), ensino médio (60,8%) e casada (45,3%). Houve maior quantidade de participantes que exercem outras atividades além de cuidar da criança (91,7%), sobretudo serviços domésticos (51,4%), com renda familiar menor que um salário (47,5%). Em sua maioria, moram até quatro pessoas na mesma casa (79,5%) e, na ausência do principal cuidador, a criança fica com os avós (38%) (Tabela 1).

A maioria das 181 crianças apresentou idade atual de 4 anos (30,4%), sexo feminino (52,2%), sendo que 86,2% sofreram algum tipo de queda no domicílio. Em relação à idade com que as crianças sofreram queda, predominou a de 2 anos (30,1%), sendo que o quarto (58,3%) foi o cômodo onde mais ocorreram esses acidentes, embora estivessem acompanhadas de algum adulto (85,3%), principalmente da mãe (67,6%) (Tabela 2).

No que concerne ao tipo de queda, predominaram queda da cama (56,4%), escorregão em piso molhado (26,3%), sofá (19,9%), bicicleta (19,9%) e rede (16,7%), sendo que as partes do corpo mais atingidas foram a cabeça (52,6%), membros inferiores (25%) e membros superiores (22,4%). A maioria das quedas não causou lesões (60,3%). Observou-se predomínio de corte e/ou laceração (16%), escoriação/raladura (14,1%) e fratura (5,8%) ocasionados pelas quedas (Tabela 3).

A maioria dos participantes (65,7%) referiu que não recebeu orientações sobre medidas de prevenção para acidentes domésticos por queda infantil. Nos casos em que os participantes do estudo foram orientados por profissionais de saúde (34,3%), referiram, principalmente, o agente comunitário de saúde (ACS) (24,2%), outro profissional (24,2%) e o enfermeiro (21%) (Tabela 4).

## DISCUSSÃO

Nesta pesquisa, verificou-se que a maior parte dos cuidadores se constituiu de mães, adultas jovens, com ensino médio e donas de casa, corroborando outros estudos^(11,17,18)^. De fato, o cuidado feminino se sobressai ao masculino, pois ainda há pouco envolvimento pessoal do pai com as crianças, embora sua participação no cuidado contribua para proteger os infantes dos acidentes domésticos^(13)^.

Estudo brasileiro realizado no Rio Grande do Sul, com homens, revelou que os serviços de saúde ainda necessitam da efetivação de estratégias para a inclusão do pai e incentivo à participação em atividades educativas, pois os mesmos se sentiram excluídos do cuidado desempenhado pelos profissionais, evidenciando que as estratégias de intervenção em saúde precisam ser (re)construídas, a fim de implementar ações que insiram o pai como participante ativo no cuidado^(20)^.

Neste estudo, a ocorrência de queda infantil em casa foi alta, como identificado em outro estudo^(11)^, indicando a necessidade de adotar supervisão ativa e contínua por parte dos adultos e priorizar intervenções voltadas para a redução da exposição infantil a tais agravos, a fim de prevenir lesões não intencionais no contexto doméstico^(21)^.

A nível global, a ocorrência de acidentes domésticos na infância demanda investimento em políticas públicas que reduzam as iniquidades sociais e a exposição aos riscos, atentando-se para os diferentes contextos nos quais as crianças estão inseridas^(22)^. Assim, considera-se necessário implementar medidas preventivas que envolvam a participação de toda a sociedade, como famílias, profissionais das áreas da saúde e educação, governos, instituições religiosas, entre outros grupos.

A respeito do perfil das crianças e a ocorrência de acidentes, nesta investigação, observou-se que houve maior frequência de queda com a idade de 2 anos, seguida da de 1 ano, ao passo que as idades de 4 e 5 anos foram menos atingidas. A literatura evidencia que a idade da criança é capaz de influenciar a ocorrência de acidentes domésticos. Desse modo, corroborando este achado, estudo de revisão integrativa revelou que, geralmente, os acidentes são menos constantes entre crianças com idade inferior a 1 ano e que o pico de ocorrência de acidentes domésticos ocorreu com crianças entre 1 e 2 anos, reduzindo para 4 anos^(22)^.

Ainda, identificou-se a mesma quantidade de ocorrência de quedas para meninos e meninas. Entretanto, estudos nacionais e internacionais mostram que o sexo masculino apresenta relação com a ocorrência de acidentes domésticos^(18,22)^, o que pode ser atribuído ao fato de que os meninos são mais expostos a situações de risco, como uso de bicicleta, brincadeiras com bola, velocípedes, patins, entre outros^(23)^.

Compreende-se que a supervisão direta de um adulto se constitui um dos fatores protetivos para impedir a ocorrência de acidentes na infância, já que o cuidado mais atencioso a uma pessoa vulnerável possibilita a identificação precoce de riscos^(13)^. Nesta investigação, porém, identificou-se que a maioria das crianças que sofreram queda em casa estavam acompanhadas de algum adulto, achado que corrobora outro estudo brasileiro^(24)^, o que evidencia a falta de supervisão atenta e o despreparo da família para identificar características e comportamentos que são específicos ao desenvolvimento infantil e que propiciam a ocorrência de acidentes. Tal fato também pode estar relacionado à ausência de ajuda nas atividades domésticas e nos cuidados com as crianças, que geralmente são realizados por uma única pessoa^(25)^.

Estudo realizado com cuidadores de crianças menores de 5 anos revelou que os acidentes domésticos são consequência de um conjunto de fatores, como o descuido do cuidador adulto e o reconhecimento (ou não) do processo de desenvolvimento da criança. Dessa forma, entende-se que as causas de tais acidentes são complexas, pois envolvem o cuidador, a criança, a família e o ambiente^(13)^. Nesse sentido, faz-se necessário compreender o crescimento e o desenvolvimento da criança, no intuito de manter um ambiente seguro de acordo com cada fase^(3)^.

Nesta pesquisa, predominaram queda da cama, escorregão em piso molhado, sofá, bicicleta e rede. Outra investigação desenvolvida no Piauí, identificou que rede alta, presença de escada ou degraus sem corrimão e saídas e passagens mantidas com brinquedos, móveis, caixas ou outros itens que possam ser obstrutivos foram associados ao risco de quedas em crianças menores de 5 anos^(17)^.

Pesquisa internacional identificou que vários fatores modificáveis foram associados a maiores chances de ocorrência de quedas de móveis de crianças com idade entre 0 e 4 anos, quais sejam: não utilizar barreiras de segurança nos cômodos da casa; deixar as crianças em superfícies elevadas; trocar fraldas em locais altos; não ensinar regras de segurança às crianças; e subir ou brincar nos móveis^(26)^.

Desse modo, estudo realizado na Nova Zelândia verificou que as modificações e os reparos de baixo custo no domicílio podem reduzir as lesões, a exemplo de corrimãos para degraus, escadas e banheiros, reparos em travas de janela, iluminação adequada, tapetes de banho antiderrapantes, entre outros^(27)^.

Ademais, verificou-se que a parte do corpo mais atingida durante a queda foi a cabeça/face (52,6%), corroborando outros estudos^(11,24,26)^, ao passo que, em outras pesquisas houve predomínio dos membros inferiores^(17)^ e membros superiores^(18)^. Acerca da natureza das lesões ocasionadas pela queda, houve predomínio de corte e/ou laceração, escoriação/raladura e fratura, convergindo com os resultados de pesquisa multicêntrica desenvolvida no Reino Unido com crianças de 0 a 4 anos, as quais sofreram quedas de móveis^(26)^.

Nesse sentido, pesquisa realizada na Austrália, com crianças menores de 3 anos, mostrou que as quedas foram a principal causa de traumatismo cranioencefálico e ferimentos na cabeça, as quais ocorreram por diferentes mecanismos, como sofá, cama, carrinho de bebê, mesa, cadeira alta, carrinho de compras, trocadores de fraldas, beliches, braços do cuidador, escadas, móveis, entre outros^(28)^.

Outro estudo mostrou que 52,8% das crianças de até 6 meses de idade são colocadas na cama dos pais para o sono noturno, mesmo na presença de berço^(29)^, o que representa riscos para quedas. Nesse sentido, pesquisa que foi realizada em Mato Grosso, revelou que, das 113 crianças estudadas, 31% sofreram algum tipo de acidente até os 6 meses de idade, com maior ocorrência de quedas (80%), principalmente da cama (45,7%)^(24)^.

A respeito das orientações sobre medidas de prevenção para acidentes domésticos por queda infantil, a maioria dos participantes referiu que não foi orientada, achado que coincidiu com outro estudo^(18)^. Nesse contexto, pesquisa realizada no Rio de Janeiro, com cuidadores, revelou desconhecimento das informações acerca da prevenção de acidentes contidas na Caderneta de Saúde da Criança, que se constitui um recurso para acompanhamento do desenvolvimento infantil^(25)^, evidenciando lacunas do conhecimento sobre a temática.

E entre os participantes que receberam orientações, a maioria relatou que foi orientada pelo ACS e por outros profissionais, seguidos do enfermeiro, em conformidade com outro estudo^(18)^. Desse modo, as práticas de educação permanente em saúde são essenciais para qualificar o ACS a fim de trabalhar a saúde da criança de modo efetivo^(30)^.

No cenário da educação em saúde, a enfermagem se destaca, porquanto tem visão holística e raciocínio crítico-reflexivo, sendo responsável pelo planejamento, implantação e avaliação das ações educativas a partir da realização de um diagnóstico situacional do público-alvo^(31)^. Portanto, uma das atribuições do enfermeiro é orientar e estimular a adoção de medidas de proteção à criança^(3)^ no âmbito das consultas de puericultura e/ou nas salas de espera dos atendimentos em saúde^(25)^, em escolas, igrejas, abrigos, áreas de esporte e lazer, entre outros.

Nessa perspectiva, entre os objetivos e metas do Plano Nacional pela Primeira Infância para a prevenção de acidentes na primeira infância, na área da saúde, estão orientação e sensibilização dos pais e responsáveis por crianças desde o início da gestação, utilizando campanhas pela televisão, folhetos, *checklists* de segurança, cartazes, reuniões em centros de saúde, nos estabelecimentos de educação infantil e nas escolas, e realização de visitas domiciliares por parte das equipes de Saúde da Família^(32)^.

Além disso, por meio da Política Nacional de Redução da Morbimortalidade por Acidentes e Violências, o Ministério da Saúde definiu como estratégia para enfrentamento deste problema diretrizes como: a promoção da adoção de comportamentos e de ambientes seguros e saudáveis; a monitorização da ocorrência de acidentes e de violências; a capacitação de recursos humanos; e o apoio ao desenvolvimento de estudos e pesquisas^(5)^.

A notoriedade dos resultados desta pesquisa ratifica a necessidade de investir esforços em intervenções educativas bem planejadas, direcionadas para os cuidadores das crianças, porquanto é imprescindível sensibilizar as famílias quanto à iminente responsabilidade de protegê-las dos acidentes domésticos, resguardando-as de lesões que podem ser evitadas por meio de mudanças de comportamentos e adoção de uma postura proativa, ao intervir de forma antecipatória nos fatores de risco, transformando a casa em um espaço seguro.

Desse modo, ao agregar conhecimento acerca da ocorrência dos acidentes domésticos por queda infantil, espera-se proporcionar subsídios para a elaboração, validação e implementação de tecnologias educativas que visem orientar as famílias quanto à adoção de práticas seguras, a fim de prevenir tais acidentes, bem como a realização de ações estratégicas contínuas voltadas para o treinamento e capacitação dos profissionais de saúde, sobretudo aqueles que acompanham as crianças nas consultas de puericultura.

Entre as limitações do estudo, destaca-se que o instrumento utilizado pontuou apenas variáveis objetivas, não possibilitando a identificação de todo o contexto do acidente, as repercussões, percepções e reações dos pais e/ou outros cuidadores diante da queda sofrida pela criança. Ainda, a ausência de um profissional intérprete de libras inviabilizou a participação de dois casais surdos.

Por se tratar de estudo com delineamento transversal, realizado em um contexto específico e pontual, no interior do estado nordestino, não é viável generalizar as interpretações para outras regiões, considerando-se as peculiaridades de cada local.

## CONCLUSÃO

As mães são as principais responsáveis pelos cuidados às crianças, além de realizar os serviços domésticos e outros trabalhos. Houve alta ocorrência de queda infantil em casa, tanto para meninas quanto para meninos, sobretudo na faixa etária de 1 a 2 anos. Ademais, a maioria das quedas ocorreu no quarto, na sala e no quintal, principalmente da cama, escorregão em piso molhado, do sofá, da bicicleta, da rede, tropeção em brinquedos e da calçada, o que ocasionou lesões como corte, escoriação, fratura, entorse, contusão, convulsão, traumatismo cranioencefálico e outras, atingindo, na maioria dos casos, a cabeça/face, membros inferiores e superiores.

Recomenda-se que os profissionais de saúde trabalhem em equipes interdisciplinares, a fim de orientar as famílias acerca das medidas de prevenção para a ocorrência de acidentes domésticos por queda infantil, ocupando os diferentes espaços sociais, como escolas, igrejas, serviços de saúde, áreas de esporte e lazer, bem como aplicando diferentes estratégias para alcançar o público-alvo, seja por meio de atividades em grupo na comunidade ou por meio de atividades individuais, – principalmente na casa onde a criança reside.

A despeito de ser um tema amplamente discutido mundialmente, sugere-se a realização de estudos voltados para a elaboração, validação e implementação de recursos educativos alternativos que estejam disponíveis para toda a comunidade típica e com deficiência, inclusive de forma lúdica e interativa, tanto para o público adulto quanto para o público infanto-juvenil, a exemplo dos materiais impressos, como – cartilhas, *folders* –, além de aplicativos, jogos, maquetes, músicas, *audiobooks* e vídeos, com vistas a garantir a acessibilidade da população às estratégias de prevenção de acidentes domésticos por queda infantil.
